# New Insight on the Formation of Sodium Titanates 1D Nanostructures and Its Application on CO_2_ Hydrogenation

**DOI:** 10.3389/fchem.2019.00750

**Published:** 2019-11-08

**Authors:** J. Noé Díaz de León, Jassiel R. Rodríguez, Joel Rojas, Yasmin Esqueda-Barrón, Luis Cardenas, Chowdari Ramesh Kumar, Gabriel Alonso-Nuñez, Sergio Fuentes-Moyado

**Affiliations:** ^1^Centro de Nanociencias y Nanotecnología, Universidad Nacional Autónoma de México, Ensenada, Mexico; ^2^Institut de Recherches sur la Catalyse et l'Environnement de Lyon - IRCELYON - UMR 5256, CNRS-UCB Lyon 1, Université de Lyon, Lyon, France

**Keywords:** CO_2_, titanates, TiO_2_, hydrogenation, greenhouse, global warming

## Abstract

The aim of this work is focused on the study of a series of non-traditional catalytic nanomaterials to transform greenhouse CO_2_ gas into added-value products. We found encouraging results of CO_2_ hydrogenation activity over sodium titanates with different morphologies. The yield to methanol increases with the increase in the Na incorporated in the nanostructures confirming the proposed mechanism. Samples were prepared at different times of hydrothermal treatment (HTT) with NaOH solutions, and these materials were labeled as Ti-nR-x with x as the hours on the HTT. HRTEM initially showed sphere-shaped nanoparticles in the TiO_2_ commercial nanopowder, increasing the HTT resulted in morphological changes in which the structures passed from nanosheets and finally to nanorods after 30 h. The X-ray diffraction and Raman spectroscopy results indicated the formation of sodium titanates such as Na_2_Ti_3_O_7_ with short Ti-O bonds and that Na begins to be incorporated into the distorted TiO_6_ crystalline structure after 5 h of HTT (until 12 wt%). The crystalline and shape transformation resulted in a significant modification on the textural properties passing from 51 m^2^.g^−1^ to 150 m^2^.g^−1^ and from a pore volume of 0.12 cm^3^.g^−1^ to 1.03 cm^3^.g^−1^ for Ti-ref and Ti-nR-30 respectively. We also observed differences in the electronic properties as the bandgap presented a blue shift from 3.16 eV on the TiO_2_ reference nano-powder to 3.44 eV for the Ti-nR-30 calcined sample. This fact coincides with the presence of a more electron-rich state of the Ti^4+^ and the formation of negative charge layer induced by the presence of Na^+^ interlayer cations detected by XPS analysis, at the same this helped us to explain the catalytic activity results.

## Introduction

Titanium dioxide is a versatile and unique material due to its optoelectronic and photochemical properties (Al-Mamun et al., 2019). Its refractive index, dielectric constant, its semiconductor bandgap have attracted widespread attention (Maheu et al., 2018). To date, many kinds of novel TiO_2_-based nano-architectures, such as nanoparticles (Dong et al., 2016), -fibers (Choi et al., 2013; Shin et al., 2016), -spheres (Cai et al., 2015; Paszkiewicz et al., 2016), -tubes (Cheng et al., 2013; Marien et al., 2016), and –rods (Bian et al., 2015; Kathirvel et al., 2016; Saber, 2016), have been prepared to modulate its electronic and catalytic properties. For instance, the photo-conversion efficiency for water splitting was duplicated, replacing the TiO_2_ nanocrystals by TiO_2_ nanowires (Khan and Sultana, 2003). On this matter, the hydrothermal method has demonstrated to be a direct way to synthesize TiO_2_ (Burungale et al., 2016; Erdogan et al., 2016; Zhang et al., 2016) and Al_2_O_3_ (Neelakanta Reddy et al., 2015) nanostructures. The hydrothermal method is often used to tailor the crystalline properties, including crystal polymorph, particle size, and shape (Kukovecz et al., 2016). One dimensional (1D) nanostructures are known for their capacity to influence the time, the temperature, the solvent, or the acid-basic conditions, all of them involved in the material synthesis (Zhang et al., 2015).

Nevertheless, the growth mechanism of nanostructures at the nanoscale as a function of those parameters is still unclear. The sequence of events for the formation of titanates in the shape of nanofibers, nanotubes, nanowires, and nanobelts have been widely proposed (Wu et al., 2006). Although, several authors coincided in the observation of lamellar structures prior the formation of 1D nanostructures and claimed that structures are formed by rolling up around the [010] direction of a single-layer nanosheet (Yao et al., 2003; Zhang et al., 2003; Wang et al., 2004). Besides, getting different morphologies was also achieved using surfactants (Sun et al., 2011) and acid promotors (Shen et al., 2008) to produce different crystal phases like anatase, rutile, and mixtures of both polymorphs. Likewise, the precursors also play a fundamental role in the nucleation, growth, and final shape of TiO_2_ nanoparticles. In this sense, it has been proposed that self-splitting of solid titanate form complex TiO_2_ nanostructures (Shen et al., 2008). Recently, we have prepared Al-doped Na-TiO_2_ nanorods varying the Al_2_O_3_/TiO_2_ ratio to be used as potential CO_2_ hydrogenation catalysts (Guzmán-Cruz et al., 2019). These results confirmed that the CO_2_ molecule could be activated over metal oxides as reported for other pure oxide systems such as Al_2_O_3_ (Pan et al., 2011), Ga_2_O_3_ (Ye et al., 2012), In_2_O_3_ (Ye et al., 2013), or TiO_2_ (Ji and Luo, 2016). We proposed that the surface mechanism for the activation of CO_2_ molecule occurs over two adjacent metal active sites (Me^*^, Al or Ti) and the presence of Na^+^ near to the neighboring of one of them, results in an active site that could interact directly with carbon from the CO_2_. Forming the -COOH followed by the stabilization of -CHO intermediate and creating a stable cyclic ring on catalyst surface.

We further analyze in this work, the growth mechanism, and the effect of Na content on the 1D titania nanorods (Ti-nR) and its application as potential CO_2_ hydrogenation catalysts. The samples were widely studied using N_2_ adsorption/desorption isotherms, X-ray diffraction (XRD), UV-vis diffuse reflectance spectroscopy (DRS), X-ray photoelectron spectroscopy (XPS) and high-resolution transmission electron microscopy (HRTEM).

## Experimental

### Chemicals

Titanium oxide (TiO_2_, 99.9%, nanopowder *d*_*p*_ = 21 nm), sodium hydroxide (NaOH, 99%), and 2-propanol (C_3_H_8_O, 99%) were purchased from Sigma-Aldrich. Millipore water (18 MΩ•cm) was used as a dissolvent. All chemicals were used as received without further purification.

### Sodium Titanates 1D Nanorods Preparation

The 1D titanates nanostructures were prepared by the hydrothermal method. In brief, 0.25 g of commercial TiO_2_ nanopowder was dispersed in 80 mL of 10 M NaOH aqueous solution during 40 min under stirring. The resultant suspension was placed into an autoclave reactor (125 mL) and thermally treated at 140°C during different aging times. The samples were labeled considering the hydrothermal treatment time (HTT) as Ti-nR-(x), where *x* = 0, 1, 5, 10, 15, and 30 h. Commercial TiO_2_ nanopowder was used as a reference labeled as TiO_2_-ref. Next, the obtained samples Ti-nR-(x) were filtered and washed with water for several times. Then samples were dried at 85°C for 1 h and finally calcined at 500°C during 4 h.

### Catalysts Characterization

The growth evolution during the formation of sodium titanates 1D nanorods was investigated by HRTEM using a JEOL 2010F microscope. The phase identification and structural analysis of the samples at different aging times of hydrothermal treatment were studied by X-ray diffraction (XRD) using a Phillips X'Pert Diffractometer, with Cu Kα (λ = 1.5405 Å) as a radiation source, at 45 kV and 40 mA. Diffraction patterns were obtained in a 2θ range from 20 to 70 (Bragg-Brentano geometry); where crystalline phases were identified with database JCPDS–ICDD 2003. Also, the presence of crystalline phases was corroborated by Raman Spectroscopy using a Horiba Xplora system interfaced with an Olympus BX41 optical microscope and a laser source of 532 nm. The evolution of absorption edge energy of the catalysts was studied using UV-Visible spectroscopy using an AVANTES Ava-spec 2048 UV–visible spectrophotometer equipped with an AvaLight-DHS light source. The absorption edge energies were obtained according to the Kubelka-Munk function, as described by Barton et al. (1999). Surface analysis of nanostructured materials was carried out by XPS using a commercial instrument Axis Ultra DLD (KRATOS). Energy positions calibration of the peak maxima was done using the binding energy (BE) of C1s peak centered at 284.6 eV. Each core level state of Ti 2p was decomposed by Doniach-Sunjic asymmetric line shapes, with an overall FWHM of 1.5 eV. The pore structure of the catalysts was investigated by N_2_ adsorption at −196°C using a Tristar II 3020 Micromeritics equipment. The surface area of the samples was determined from the Brunauer–Emmett–Teller (BET) equation and the pore volume from the total amount adsorbed at a relative pressure near unity. The pore size distribution was analyzed using the Barrett–Joyner–Halenda (BJH) method.

### Activity Measurements

All the synthesized catalysts were evaluated for the CO_2_ hydrogenation in a microflow reactor in the temperature range of 300–340°C and under atmospheric pressures. In a typical experiment, 0.1 g of the catalyst sample was loaded into the reactor and heated up to the desired temperature with a ramp of 5°C min^−1^. Once the temperature was attained, a gas mixture of H_2_ and CO_2_ (3:1) was passed through the reactor with flow of 40 mL min^−1^ (space velocity ~15,000 h^−1^), and the outlet of the reactor was connected to an online GC-TCD-FID Agilent SP1 equipped with a series of seven columns, generally used in special refinery gas application. CO_2_ hydrogenation experiments were carried out at three different temperatures viz. 340°, 300°, 320°, and 340°C for 5 h, and the products were analyzed every 30 min. Under steady-state conditions we considered at least 6 stabilized samples to obtain an average conversion ($\bar{x}$) value. The $\bar{x}$ where obtained as indicated in Equation 1:

(1) $$\begin{array}{r} \bar{x}=\frac{C_{CO2}^{0}-C_{CO2}^{T{}^{\circ}C}}{C_{CO2}^{0}} \end{array}$$

Where $C_{CO2}^{0}$ is the initial concentration at RT and $C_{CO2}^{T{}^{\circ}C}$ is the concentration after reaction at temperature T°C.

Considering that our conversions resulted below 10%, we could assume differential conditions and therefore the reaction rates at different temperatures could be calculated using the next equation:

(2) $$\begin{array}{r} r_{CO2}=\frac{\bar{x}*Q_{CO2}^{0}}{m_{cat}} \end{array}$$

Where $Q_{CO2}^{0}$ is the molar flow of CO_2_, and m_cat_ is the catalysts mass used in the specific catalytic activity test. The yield was obtained using the ratio $C_{i}^{T{}^{\circ}C}$/ $\left(\underset{i}{\sum }C_{i}^{T{}^{\circ}C}+C_{CO2}^{T{}^{\circ}C}\right)$ where $C_{i}^{T{}^{\circ}C}$ is the concentration of products in the reactor effluent.

## Results

### Morphological Effect of Na Into the Nanostructures by HRTEM

**Figure 1:**
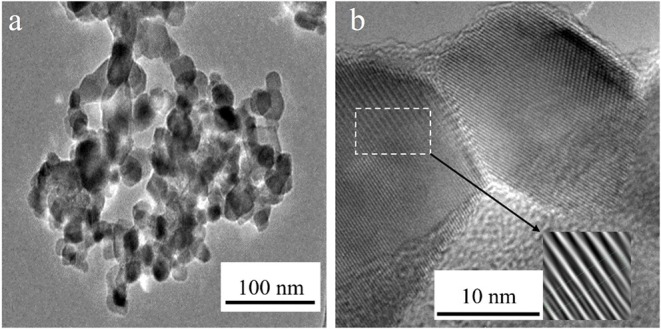
**(a)** Typical TEM micrograph obtained for the Ti-ref and **(b)** HRTEM micrograph for the nanoparticles in the Ti-ref and an IFFT image as an inset.

Figure 1 presents the characteristic nanoparticles for the TiO_2_-ref with an average particle size of 21 ± 1 nm. In Figure 1 is also possible to see the inverse fast Fourier transform (IFFT) used in the calculation of interplanar distances (d_s_) included as an inset. The values for the d_s_ resulted in 3.4 Ȧ, this value is closed to that reported (3.51Ȧ) for the plane (1 0 0) of anatase according to the 01-084-1286 file of ICCD 2003. Other zones with no observable planes are present in the sample suggesting that the reference material is composed of Anatase and another crystallographic phase. This will be further confirmed with the XRD analysis.

**Figure 2:**
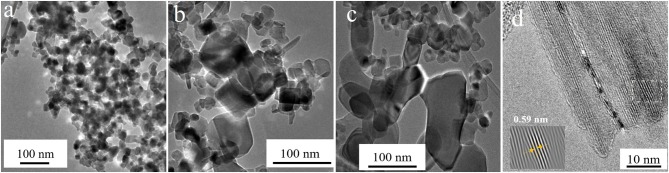
TEM micrographs correspondent to **(a)** Ti-nR-0, **(b)** Ti-nR-1, and **(c)** Ti-nR-5. **(d)** HRTEM micrograph for a nanosheet in Ti-nR-5 sample and a IFFT as an inset.

The typical micrographs acquired for the Ti-nR-0, Ti-nR-1, and Ti-nR-5 are shown in Figure 2.

Initially, the nanoparticles that were treated with the alkaline solution for 40 min, but without hydrothermal treatment did not present any morphological changes as shown in Figure 2a. For the Ti-nR-1, the presence of the original nanoparticles is also observed along with some layered nanosheets of various sizes (Figure 2b). In the case of Ti-nR-5, the presence of raw nanoparticles was barely observed, and large titania layered nanosheets became predominant all over the sample (see Figure 2c). The chemical attack from the alkaline treatment induces the peeling of these observed layers from the nanoparticles, and the time of hydrothermal treatment determines the formation of this new morphology. As described in other reports, the formation of layered nanosheets might be the initial step in the route to the formation of all layered materials, as the TiO_2_ nanotubes (Bavykin et al., 2004) or the multiwalled carbon nanotubes (Viculis et al., 2003) after splitting.

Nevertheless, the exact sequence of events is still unclear, and several mechanisms for the subsequent formation of 1D nanostructures have been proposed (Viculis et al., 2003; Bavykin et al., 2004; Das et al., 2008; Lee et al., 2014). In general, is suggested that the NaOH begins disturbing the initial crystalline structure of the titania nanoparticles, then the basic unit of the structure (octahedral units) rise hydroxyl bridges with Ti ions and finally growing in a preferential direction the sheets are formed (Wang et al., 2004). Indeed, as seen in Figure 2d, the single nanosheet exhibit planes in one direction, the interplanar distances observed of around 0.59 nm are related to the (−2 0 1) plane of the sodium titanates, following the 00-013-0589 file of ICCD 2003. This result may suggest that the intercalation occurs not only between TiO_2_ octahedral units but also including the Na ions. The XRD analysis will provide us further information about the resulting crystalline structure on samples after the thermal treatment.

**Figure 3:**
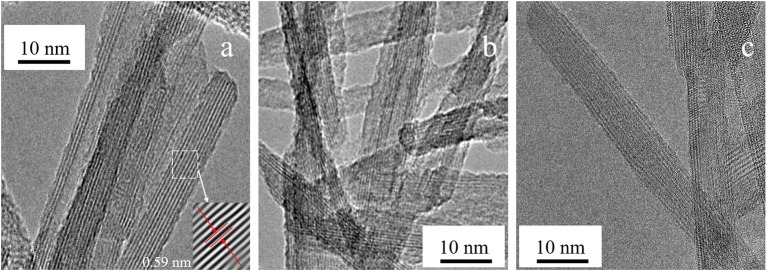
HRTEM for the **(a)** Ti-nR-10, **(b)** Ti-nR-15, and **(c)** Ti-nR-30.

The Figure 3 shows some HRTEM micrographs for the calcined Ti-nR-10 and Ti-nR-30 samples. In Figure 3a is possible to observe a nanosheet that seems to begin to be separated into well-defined sections. With the increase in HTT, the sheets lose wholeness, and the rods segregate from each other, and finally after 30 h (see Figure 3b), the 1D nanorods are clearly defined, and nanosheets or nanoparticles are no longer observed.

### X-ray Diffraction

The X-ray diffraction patterns for the calcined Ti-nR-x series are provided as Supplementary Information 1. The TiO_2_-ref sample exhibited 75% of anatase and 25% of rutile crystalline phases according to the Rietveld analysis, following the files 01-084-1286 and 01-078-1508–0551 of database ICCD 2003. The simulated scan patterns for the anatase and rutile phases are presented along with the patterns for the Ti-nR-0 and Ti-nR-1 samples in Figure S1A. These two samples presented the same diffraction peaks and intensities as the TiO_2_-ref samples; the Rietveld analysis confirmed this statement since it only showed slight differences in their composition. This fact revealed that the sodium titanates are not formed at low hydrothermal treatment time. The diffraction patterns for the samples with longer HTT are shown in Figure S1B. It is possible to observe that the patterns exhibit peaks with low intensity compared with the reference and samples in Figure S1A. All samples presented diffractions at ca. 10.5°, 24.7°, 29.3°, 33.2°, 44°, 48°, and 67° 2θ degrees. These peaks are consistent with the formation of sodium titanates, such as Na_2_Ti_3_O_7_ and Na_2_Ti_3_O_13_ (Wang et al., 2015). Although the peaks at 29.3° and 48° were attributed to the formation of small quantities of the rutile phase. The diffractions at 10.5°, 24.7°, and 44° 2θ degrees corresponding to the crystalline diffractions of Na_2_Ti_3_O_7_, while peaks at 33.2° and 67° 2θ degrees are related to the Na_2_Ti_3_O_13_ phase. We can see that the peaks of the respective titanates increase with the HTT, especially the Na_2_Ti_3_O_13_ phase. Average crystallite sizes of calcined materials are listed in Table 1, calculated using the Scherrer's equation. The results showed an almost linear behavior of crystallite size with an increase in the HTT.

**Table 1:** Crystal size estimation and textural properties for the Ti-nR-x series.

**Sample**	**Crystallite size nm**	**S_**BET**_ m^**2**^ g^**−1**^**	**Vp cm^**3**^ g^**−1**^**
TiO_2_-Ref	23.1	51	0.12
Ti-nR-0	25.2	50	0.15
Ti-nR-1	22.8	53	0.15
Ti-nR-5	8.2	104	0.55
Ti-nR-10	5.1	108	0.64
Ti-nR-15	4.0	121	0.71
Ti-nR-30	3.8	150	1.03

### Textural Properties

The Ti-nR-x catalysts were analyzed by nitrogen physisorption technique. The isotherm and pore size distributions plots are provided in the supplementary information as Figure S2. The textural properties of the Ti-nR-x series are included in Table 1. As observed, a clear variation in the surface area was detected depending on the HTT. The reference sample presented only 51 m^2^.g^−1^ and a low pore volume of 0.12 cm^3^.g^−1^. As expected, Ti-nR-0 and Ti-nR-1 samples displayed almost the same textural properties as the reference sample. When the chemical attack of NaOH begins to be significant over the TiO_2_ nanoparticles, and the formation of sodium titanates are observed, the surface area increased almost two times (104 m^2^.g^−1^). Sample with 10, 15, and 30 hours of hydrothermal treatment exhibited 108, 121, and 150 m^2^.g^−1^, respectively. Although the difference in surface area between Ti-nR-10 and Ti-nR-15 samples is only 4%, the Ti-nR-30 presented an increase near to 30% with respect to the same sample (Ti-nR-10). These changes described are related to the progressive formation of the new nanostructures with more area than the solid nanoparticles in the raw material. The pore volume also presented the same trend as the surface area shifting from 0.12 to 1.03 cm^3^ g^−1^ in TiO_2_-ref and Ti-nR-30 samples, respectively. The isotherms plot and pore size distributions are provided as Supplementary Information.

### Diffuse Reflectance UV-vis Spectroscopy

**Figure 4:**
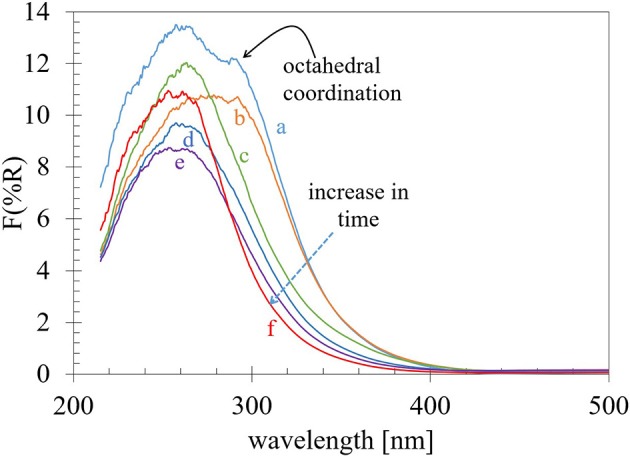
UV–vis diffuse reflectance absorption spectra for the **(a)** TiO_2_-ref, **(b)** Ti-nR-1, and **(c)** Ti-nR-5, **(d)** Ti-nR-10, **(e)** Ti-nR-15, **(f)** Ti-nR-30 samples.

The UV–vis diffuse reflectance absorption spectra for the Ti-nR-x series are shown in Figure 4. An evident variation on the absorption edge was observed due to the different HTT. In the TiO_2_-ref sample, at least two absorptions can be observed: a strong band centered at 262 nm and a small absorption shoulder with its maximum c.a. 292 nm. The first one is usually related to Ti^4+^ in tetrahedrally coordinated sites (TiO_4_), and the second one is assigned to Ti atoms in octahedral coordination (TiO_6_) (Capel-Sanchez et al., 2000). As shown in Figure 4, the intensity of the absorption band related to the Ti octahedrally coordinated atoms decreases considerably with the increase in the HTT. Here it is worth to mention that in principle, the hydrothermal treatment to the TiO_2_ nanopowder (TiO_2_-ref) drive into the formation of sodium titanates as the XRD results exhibited. Then, the formation of Na_4_TiO_4_, which is an ionic solid formed by isolated TiO_4_ tetrahedrons (Doeff et al., 2014), could be inducing the observed blue shift in the optical absorption band edge. Nevertheless, as the XRD results indicated, Na_2_Ti_3_O_7_ and Na_2_Ti_3_O_13_ crystalline structures were formed, and both consist of TiO_6_ octahedrons connected through edges or corners to form layered structures where the Na ions stay between layers (Doeff et al., 2014). Then the observed shift is a combination of the decrease in the quantity of TiO_6_ octahedra connected each other and the intercalation and corresponding increase in the insulator character. Then we will assume that our material is a combination of different titanates namely few Na_4_TiO_4_, Na_2_Ti_3_O_7_, and Na_2_Ti_3_O_13_.

The evaluation of the edge energy (E_g_) bandgap usually is obtained from the UV–vis diffuse reflectance absorption spectroscopy. In the literature, there are several reports of E_g_ values for titania, but as the values are obtained according to the type of transition band considered, directly or indirectly and depending on absorbance or reflectance data, the E_g_ values can strongly differ from one author to the other (López and Gómez, 2012). The most accepted method, for determining the band-gap energy values of semiconductors is by plotting a function of reflectance absorption such as the Kubelka–Munk equation (Equation 1) given by F(R)$=\frac{{\left(1-R\right)}^{2}}{2R}$ without considering electronic transitions or [F(R).h*v*]^1/n^ with n as 2 for an indirect allowed transition or $\frac{1}{2}$ for a direct allowed transition, vs. the photon energy and extrapolating the linear part of the rising curve to zero (Barton et al., 1999; Reddy et al., 2002; López and Gómez, 2012).

**Figure 5:**
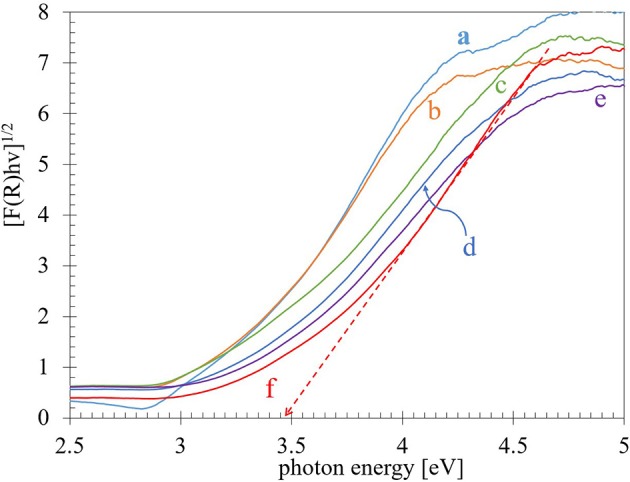
Kubelka-Munk function plotted versus photon energy for each sample **(a)** TiO_2_-ref, **(b)** Ti-nR-1, and **(c)** Ti-nR-5, **(d)** Ti-nR-10, **(e)** Ti-nR-15, **(f)** Ti-nR-30 samples.

Plotting different functions of UV-vis diffuse reflectance, it is possible to obtain E_g_ values, which highly differ from each other until 0.4 eV. The obtained values were collected in Table 2. For comparison with our results, the widely reported intrinsic bandgap absorption for pure TiO_2_ anatase is in the range between 3.15 and 3.2 eV (Yu et al., 2009; Ji and Luo, 2016; Maheu et al., 2018). Also, the XRD-Rietveld analysis results revealed that our TiO_2_-ref nanopowder is 75% anatase and 25% rutile. Therefore, our E_g_ values considering *n* = 2 for an indirectly allowed transition in the KM transformation leads to similar values to those reported in the literature for this solid. Then the values obtained for the Ti-nR-x series will be further discussed considering only the indirect allowed transition (*n* = 2) (Figure 5). An increase in the E_g_ values with the hydrothermal treatment was observed from 3.16 eV in the TiO_2_-ref sample to 3.19, 3.23, 3.29, 3.35, and 3.44 eV for Ti-nR-1, Ti-nR-5, Ti-nR-10, Ti-nR-15, and Ti-nR-30 samples respectively. As mentioned, this increase in the insulator character is due to the presence of the Na in the TiO_2_ matrix.

**Table 2:** Edge energy for the Ti-nR-x series obtained from different treatments of the UV-vis diffuse reflectance data.

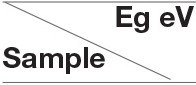	**F(%R)**	**(F(%R)xhv)^**1/2**^**	**(F(%R)xhv)^**2**^**
TiO_2_-ref	3.48	3.16	3.81
Ti-nR-1	3.51	3.19	3.86
Ti-nR-5	3.65	3.23	3.98
Ti-nR-10	3.67	3.29	4.11
Ti-nR-15	3.72	3.35	4.15
Ti-nR-30	3.89	3.44	4.24

### Raman Spectroscopy

**Figure 6:**
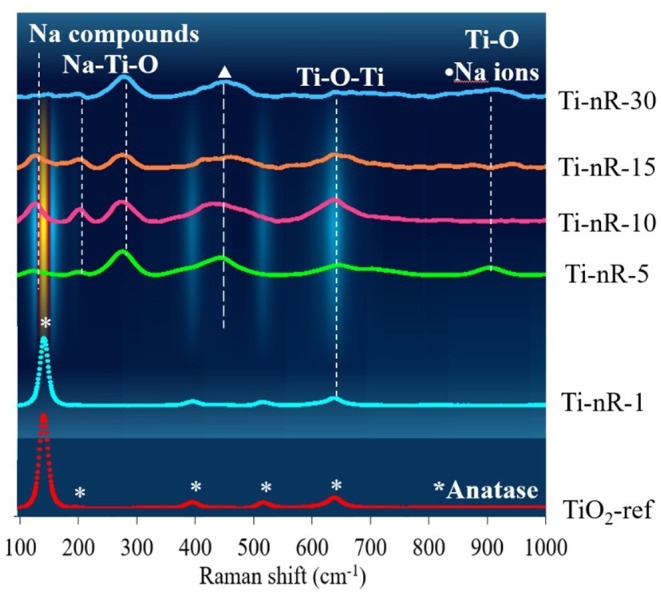
Raman spectra of Ti-nR-x series as a function of the hydrothermal treatment time.

Figure 6 shows the Raman spectroscopy analysis obtained for the Ti-nR-x series. The Raman spectra obtained for the commercial TiO_2_ reference and the Ti-nR-1 exhibited the same six active modes related to the TiO_2_ anatase crystalline phase with space group *I*4_1_/*amd* (Ma et al., 1998). The anatase phase typically exhibited a tetragonal structure with vibration modes in 144 cm^−1^ (E_g_), 197 cm^−1^ (E_g_), 399 cm^−1^ (B_1g_), 513 cm^−1^ (A_1g_), 519 cm^−1^ (B_1g_), and 639 cm^−1^ (E_g_) (Balachandran and Eror, 1982; Qian et al., 2005). Changes in the vibrational modes of mentioned TiO_2_ crystalline structure were observed after 5 h of hydrothermal treatment, in good agreement with the XRD results presented in section X-ray diffraction (*vide supra*). The chemical attack of NaOH during the synthesis procedure derived in the formation of sodium titanates (Na_2_Ti_3_O_7_) which is reflected in a significant loss of symmetry.

The Raman spectra for the Ti-nR-5, Ti-nR-10, Ti-nR-15, and Ti-nR-30 catalysts exhibited low intensity and wider bands c.a. 130, 204, 278, 445–462, 637–646, and 902–910 cm^−1^. From those mentioned Raman active modes, the bands at 123 cm^−1^ thas been assigned to (A_g_) αNa2, the band in 201 cm^−1^ to (B_g_) τO_1_-Ti_3_-O_2_, meanwhile the band at 277 cm^−1^ to τO_5_-Ti_2_-O_6_ with stretching mode related to Na-O-Ti, and finally the bands in 449 and 643 cm^−1^ to σO5-Ti1-O4 in Ti-O-Ti stretching and edge shared TiO_6_ vibrational modes (Zhang et al., 2010; Silva et al., 2018). All vibrational modes of the Ti-nR-x series are listed in Table S1 provided as supplementary information. The phase transformation observed for samples with thermal treatment times ≥5 h confirmed the intercalation of Na into the layers of TiO_6_ octahedral species. Even when the nanoparticles formed could make the surface more predominant and increase the surface-volume ratio, the intensity of the spectra was very low, suggesting that phonon confinement, and structural defects were not observed due to the presence of intercalated Na (Ilie et al., 2017).

### Inductively Coupled Plasma (ICP)

ICP measurements (Table S2) confirmed the variation in the Na with the hydrothermal treatment time. The atomic weight composition for the titanates (Na_2_Ti_3_O_7_) phase is related to a sodium stoichiometric bulk concentration of around 15. wt % while for the Ti is near to 47 wt %. Therefore, following the results enlisted in Table S2 from material Ti-nR-5, the composition seems to be stabilized on average at 11.9 wt % Na and 43.8 wt % for Ti. These values confirmed the formation of sodium titanates as exhibited by XRD and Raman spectroscopy results.

### X-ray Photoelectron Spectroscopy

**Figure 7:**
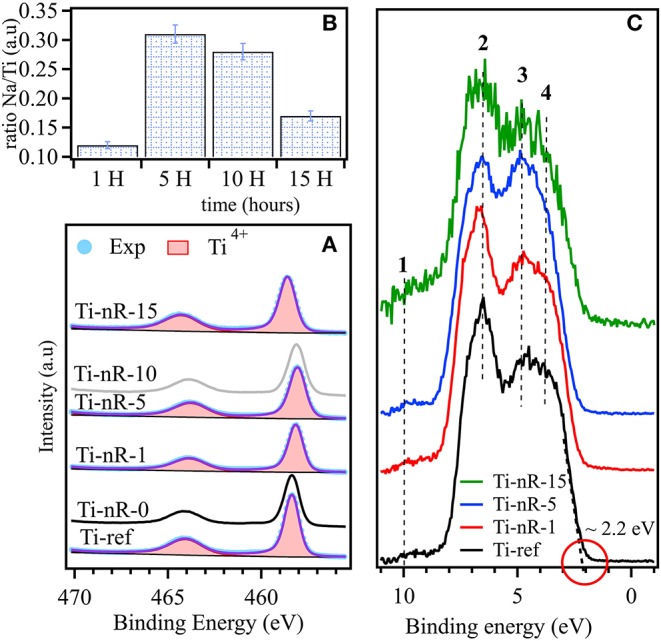
**(A)** Na/Ti XPS chemical surface ratio, **(B)** XPS spectra of Ti 2p for Ti-ref, Ti-nR-0, Ti-nR-1, Ti-nR-5, Ti-nR-10 and Ti-nR-15. **(C)** XPS valence band corresponding to Ti-ref, Ti-nR-1, Ti-nR-5, and Ti-nR-15.

XPS analysis was used in order to establish the influence of the hydrothermal reaction time with the presence of Na on the surface. The surface atomic quantification provided the increase of the Na/Ti ratio as a function of the hydrothermal treatment time (HTT), as seen in Figure 7A. XPS chemical surface quantification revealed that after 10 H, Na incorporation is attained. In this context, at least two interpretations appear possible. By one hand, after 10 hours under HTT a hysteresis loop on the surface may be established so that the adsorption sites are lost. On the other hand, the hydrothermal process stabilizes after around ten hours and does not allow incorporate more Na into the nanoparticles.

Figure 7B shows the XPS spectra of the Ti 2p core level obtained from Ti-ref, Tin-R-0, TinR-1, TinR-5, TinR-10, and TinR-15. The Ti 2p state was fitted using a Doniach Sunjic line-shape. The choice of this function is based on strong electron-hole interaction responsible for the asymmetric character of this state. XPS analysis on Ti 2p core level (Figure 7B) revealed the presence of Ti^4+^ for all the samples, Ti 2p_3/2_ is located at ~458.3 eV this position is consistent with anatase and rutile phases (Georgakopoulos et al., 2019). Trivalent Ti^3+^ is an unstable phase at the surface of TiO_2_ then is difficult to observe by XPS. Therefore, a valence band (VB) analysis was carried out in order to get insight into occupied electronic states.

Figure 7C exhibits the XPS-VB obtained from Ti-ref, TinR-1, TinR-5, and TinR-15. For all spectra, the baseline was corrected using a Tougaard background function. The VB maximum (EVB_max_) was obtained at ~2.2 eV for all the samples following the method developed by Maheu et al. (2018). At low binding energy it was detected four significant contributions labeled from **1** to **4**, as proposed by Fleming on TiO_2_/Si(100) (Fleming et al., 2007). The electronic state labeled in Figure 7C as **1** is located at ~11 eV this state is normally associated with OH groups (Sanjinés et al., 1994). The other three states (**2**, **3**, and **4**) could be interpreted using molecular orbital approximations (Goodenough, 1971). Highlighting the work of Asahi et al. (2000), the electronic states **2** and **3** could be associated with the hybridized bonding orbitals meanwhile the contribution **4** is related to O 2p orbitals located at ~1 eV below the energy of maximum value of VB (EVB_max_) this contribution arises from the hybridized non-bonding orbitals. The position of occupied states in the VB may not be sensitive to HTT or Na insertion. Therefore, the energy variations in the bandgap are coming from the unoccupied states. Our UV-vis results in section Diffuse reflectance UV-vis spectroscopy, gave us light about this matter since the spectra for the Ti-ref and Ti-nR-0 presented octahedral and tetrahedral coordination. Then, after the hydrothermal treatment, it seems that the octahedral coordination contribution was drastically reduced, remaining only, which appears to be the tetrahedral coordination. Nevertheless, this phenomenon seems to be only an apparent effect provided by Na intercalated ions. In any case, the shift observed is further related to a change in coordination and not to the presence of Ti^3+^ species under the experimental conditions of XPS analysis (non-reducing environment).

### CO_2_ Catalytic Hydrogenation

**Figure 8:**
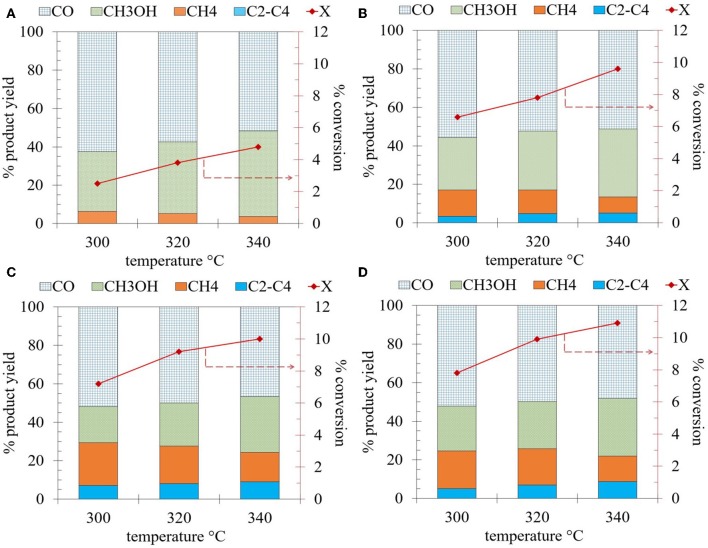
Conversion of CO_2_ and yield of products over the Ti-nR-x series catalysts: **(A)** Ti-nR-5, **(B)** Ti-nR-10, **(C)** Ti-nR-15, and **(D)** Ti-nR-30.

The CO_2_ hydrogenation activity measurements were performed in the temperature range of 300–340°C over Ti-nR-x catalysts, and the results are presented in Figure 8. The differential reactions rates of the catalytic hydrogenation of CO_2_ to produce CO, CH_4_, CH_3_OH, and C_2_-C_4_ hydrocarbons are presented in Table S3. Under the experimental conditions used in this work, we observed all the products listed before. Among them, the production of methanol would be preferred because of its industrial interest (László et al., 2019).

Initially, and for comparison, the Ti-ref sample was also tested, and its activity is provided in the supplementary information as Figure S3. The Ti-ref (raw material) exhibited about 6% of CO_2_ conversion at 300°C and as expected the conversion increased to around 8 and 9% with an increase in temperature of 320°C and 340°C, respectively. In the case of the yield, the main product, irrespective of the reaction temperature, resulted in carbon monoxide as reported for similar materials (János et al., 2019). However, the CO selectivity decreased from 68.5 to 61.3% with an increase in the reaction temperature from 300–340°C as well the C_2_-C_4_ product yield decreased from 18.2 to 4.5%. Both changes favored at the same time a drastic increase in the yield to methanol from 2.1 to 25% in the same range of temperatures.

In the case of the Ti-nR-5 catalyst, lower CO_2_ conversion was observed (max. of 4.8% at 340°C) in comparison to the Ti-ref sample (Figure 8A). However, the yield toward methanol considerably increased to 44.7%. The yield toward CO and CH_4_ resulted in 51.7% and 3.6%, respectively, and no C_2_-C_4_ hydrocarbons were observed for this catalyst at 340°C. The lower conversions for the Ti-nR-5 sample might be due to the incorporation of the Na ions into the nanostructures, which resulted in a wide variation in its structural, morphological, and electronic properties in comparison to the raw TiO_2_ nano-powder material. While, in the case of Ti-nR-10, Ti-nR-15, and Ti-nR-30 catalysts (increase in HTT time, Figures 8B–D), at all the reaction temperatures slightly high CO_2_ conversions were observed in comparison to the Ti-ref sample and the highest conversion was achieved at 340°C. The conversion of CO_2_ could be correlated to the hydrothermal treatment time. As seen, with the increase in the HTT larger is the CO_2_ conversion, which reaches a maximum of 10.9% in the case of Ti-nR-30 catalyst. The conversions observed are in the range of other reported values for similar materials (Wang et al., 2017; Guzmán-Cruz et al., 2019). Although the yield to methanol decreased with increase in the HTT time. The methanol yield was near to 35% in the case of the Ti-nR-10 catalyst and remains constant (about 29-30%) for the Ti-nR-15 and Ti-nR-30 catalysts. We can conclude from this results that the best catalytic performance in the CO_2_ hydrogenation is provided by Ti-nR-30 catalyst because the conversion resulted near to 11% at 340°C and at the same time showing an excellent yield toward methanol (30%).

## Discussion

The electronic properties, the high surface area, and its cation exchange capacity become the 1D titanates nanostructures of paramount importance in the catalysis field. There is the possibility to use it for a wide variety of applications such as supports for Au single atoms and clusters as well for other metals, for the photocatalytic transformation of CH_4_ and CO_2_ hydrogenation among several others (János et al., 2019). In this work, we systematically analyze the genesis, formation mechanism, and the general physicochemical and catalytic properties of 1D sodium titanates. For example, we observe that increasing the time of thermal hydrotreatment of the commercial titania nano-powder several changes occur in its morphology, in its crystalline structure, and its textural and electronic properties. The mechanism of nanostructures formation by hydrothermal treatment with NaOH of TiO_2_ nanoparticles is delamination first to form nanosheets of titanates, and then a reassemble by peeling and scrolling goes forward in nanotubes (Lee et al., 2014).

Nevertheless, we observed that the structural changes and shaping are hydrothermal treatment time-dependent. Indeed, we detected the formation of the nanosheets in short times (Ti-nR-5), as displayed in Figures 2b–d. Afterward, the scission of nanosheets conducted directly to the 1D type nanorods after 30 h (Ti-nR-30). We did not observe the formation of any scrolled structure or tube, as mentioned by literature, at least not until the maximum time of hydrothermal synthesis (30 h).

The nanosheets found in TEM analysis indicated that the crystalline phase in it is different from that observed in the precursor sample. The intercalation of Na ions between the unitary cells of TiO_2_ derived in the increase in the interplanar distances. Although, the Na content does not increase linearly with the thermal treatment time as we observed via ICP measurements and confirmed by XPS analysis. The chemical attack of NaOH to the structures rather induce the scission of the nanosheets, keeping low the Ti/Na ratios (Table S2), confirming that low Ti/Na ratios could be related to titanates structures (Wu et al., 2018; Liu et al., 2019), same that was confirmed with the XRD and Raman analyses, as exhibited in Figure S1 and Figure 7, respectively. In the case of the surface area, the increase was almost linear after 5 h of thermal treatment time passing from 51 m^2^g^−1^ in the reference sample to 150 m^2^g^−1^ in the case of the Ti-nR-30 sample (see Table 1). However, this increase in the general textural properties is not only related to the morphological shape transformation but also the formation of interconnected networks of the 1D nanorods (Díaz de León et al., 2014; Guzmán-Cruz et al., 2019).

Several authors reported that Ti^3+^ and oxygen vacancy are related to defect sites in titanates making it generally more active in applications that need electron donor behavior (Wang et al., 2011), in this sense, we carried out the XPS analysis to confirmed the absence of Ti^3+^ in all samples. The XPS analysis revealed the presence of one Ti 2p_3/2_ species at 458.6 eV; this position is consistent with Ti^4+^ in anatase and rutile phases but not with the Ti^3+^. We observed slight variations on the Ti 2p_3/2_ position to lower binding energies with the increase in the HTT. This observation could be related to the presence of a more electron-rich state of the Ti^4+^ and the formation of a negative charge layer induced by the presence of Na^+^ interlayer cations (Meroni et al., 2017).

Related materials have been tested for the CO_2_ thermal hydrogenation (Guzmán-Cruz et al., 2019; János et al., 2019; László et al., 2019) exploring the reverse water gas shift reaction as a first approximation of reaction mechanism. In it, the CO_2_ dissociates in CO and O over the more metallic sites. In this case, the CO, recently formed, desorbs from the catalytic site and the O form water with another molecular H_2_ available in the proximity. Nevertheless, our experimental results not only reported the formation of CO but also the production of methane and methanol on the Ti-nR-x series. Then the mechanism for the hydrogenation of CO_2_ molecule over the Ti-nR-x catalyst could be adopted from that one reported recently for similar materials such as AlTi-nR-x (Guzmán-Cruz et al., 2019), and ZnO–ZrO_2_ catalysts (Wang et al., 2017). In those works, it was reported that the H_2_ reduction ambient removes first the surface hydroxyl groups and lattice oxygens forming water. Afterward, the CO_2_ interacts with the free electrons of the more electron-rich state of the Ti^4+^ induced by the presence of Na^+^ forming -COOH on catalysts surface. Further hydrogenation of this formates completes the plausible mechanism.

## Conclusions

In this work, TiO_2_-sodium titanates nanorods (Ti-nR-x) have been successfully prepared by means of the hydrothermal method at various intervals of time. The general characterization results revealed the transformation and growth mechanism of the sodium titanates 1D nanorods. We observed the evolution of the crystalline phases and the incorporation of sodium ions into the tetrahedral unit cell to form the 2D nanosheets. This Na intercalation promotes the subsequent formation of the 1D nanorods by its scission. Electronically, the shift to lower binding energies was provided by a more electron-rich state of the Ti^4+^ species. The results of the optical characterization showed that the energy gap increases with the aging time of the samples confirming the intercalation of the Na ions resulting in the observed decrease in the conductor character. The catalytic activity measurements revealed an almost linear function with the hydrothermal treatment time of synthesis. The intercalation of the Na^+^ ions seems to provide an excellent electronic ambient in which the H_2_ and CO_2_ molecules might be activated on adjacent metal active sites (Ti^*^) produced under experimental conditions through water removal from the surface. We conclude that such 1D type sodium titanates with nanorods shape are suitable for the intended CO_2_ hydrogenation to decrease the greenhouse effect and produce value-added products, it is also clear that its properties could be of great interest in other fields as photocatalysis, sensors, dye-sensitized solar cells among several others.

## Data Availability Statement

The datasets generated for this study are available on request to the corresponding author.

## Author Contributions

JD: original writing, review, and editing. JRR and JR: synthesis and general characterization. YE-B: Raman analysis and discussion. LC: XPS analysis, writing, and discussion. CR: writing and discussion. GA-N: discussion. SF-M: conceptualization and supervision.

### Conflict of Interest

The authors declare that the research was conducted in the absence of any commercial or financial relationships that could be construed as a potential conflict of interest.
