# Supplementary material for: New Insight on the Formation of Sodium Titanates 1D Nanostructures and Its Application on CO2 Hydrogenation
Source: Front Chem. 2019 Nov 8;7:750. doi: 10.3389/fchem.2019.00750 (PMC6856218; doi:10.3389/fchem.2019.00750)
Supplement: Supplementary file 1 [file Data_Sheet_1.docx]

**New insight on the formation of Sodium Titanates**

**1D nanostructures and its application on CO_2_ Hydrogenation**

J.N. Díaz de León^*,1^, J. R. Rodríguez^1^, J. Rojas^1^, Y. Esqueda-Barrón^1^, L. Cardenas^2^, Ch. Ramesh-Kumar^1^, G. Alonso-Nuñez^1^, S. Fuentes^1^

**Fig. S1** A Right)) X-ray diffraction patterns for the TiO_2_-ref, b)Ti-nR‑0 and c)Ti-nR-1 samples along with simulated patterns for anatase and rutile phases. B left) Diffraction patterns for: d) Ti-nR‑5, e)Ti-nR‑10, f)Ti-nR‑15, g)Ti-nR-30 samples and simulated patterns for Na_2_Ti_3_O_7_ and Na_2_Ti_3_O_13_


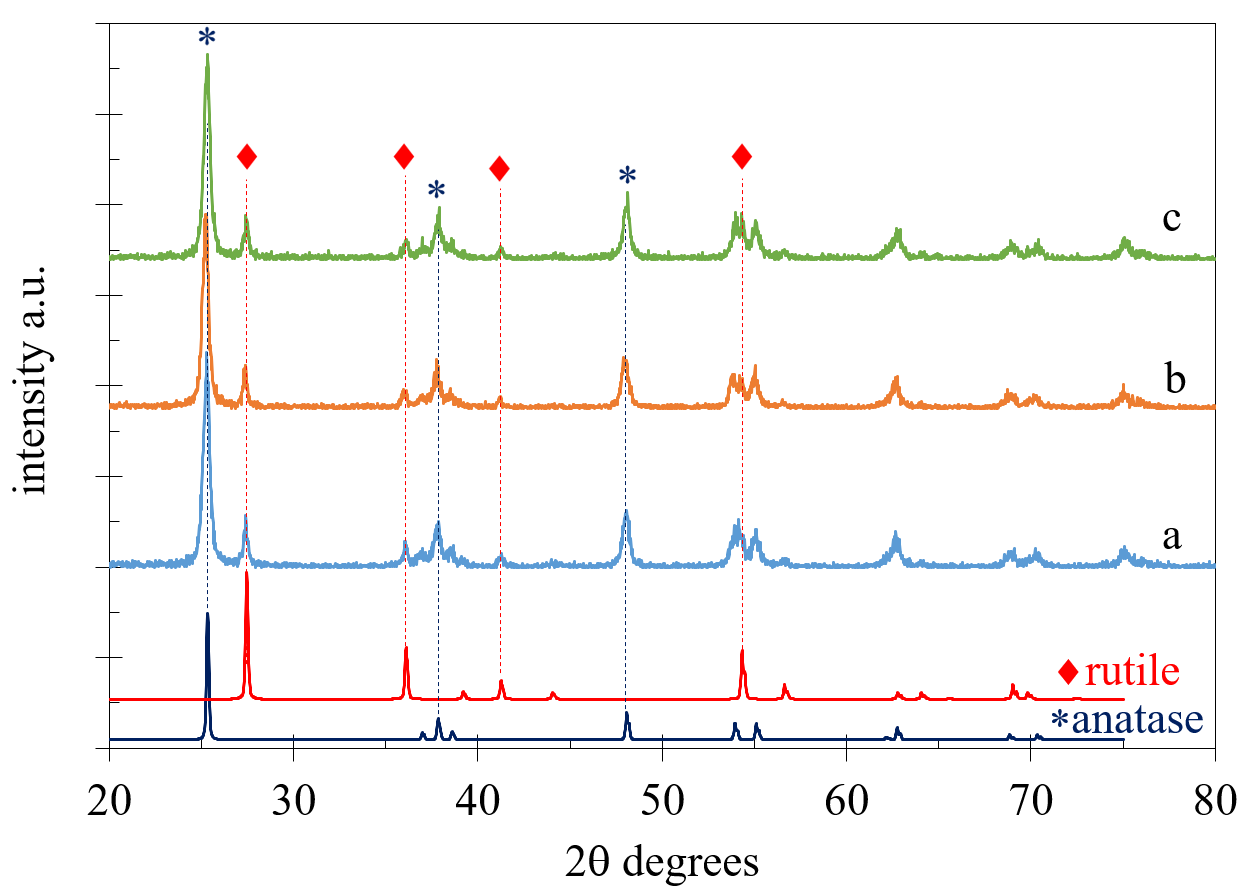

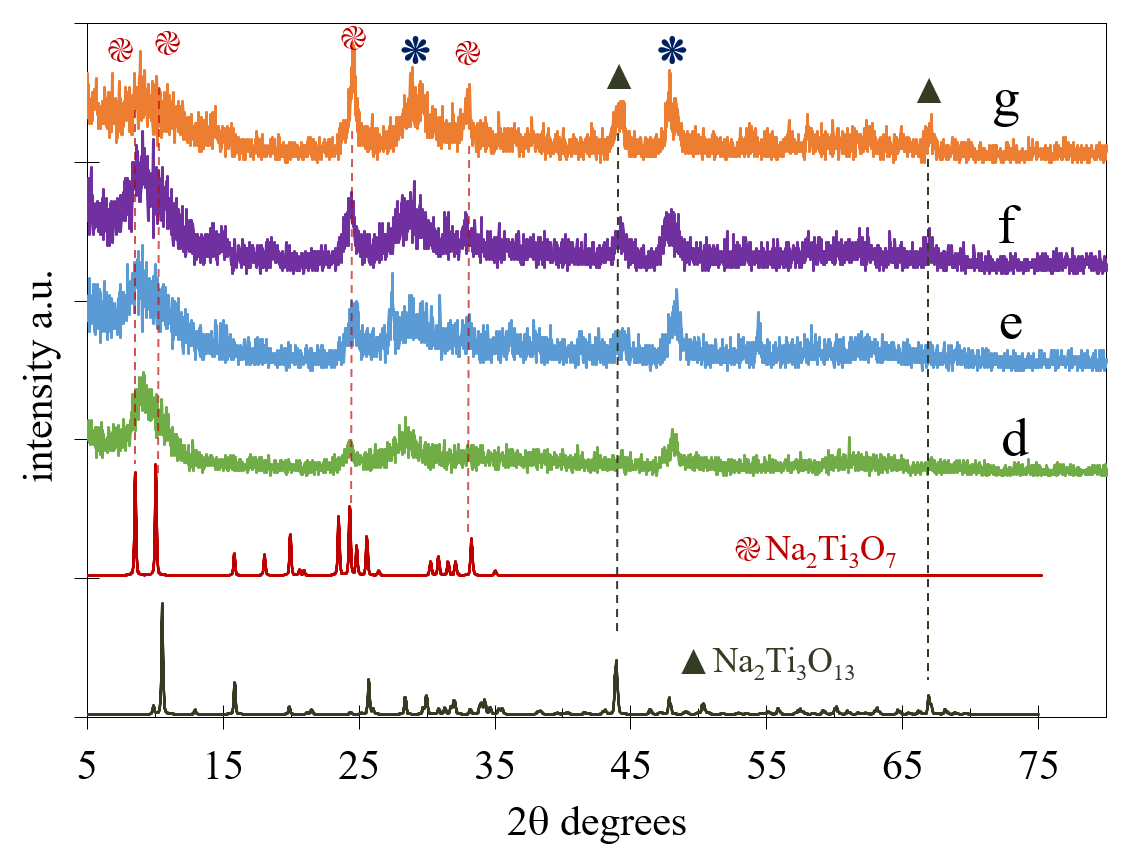


**Fig S2.** Nitrogen physisorption analysis (A) adsorption-desorption isotherms and (B) pore size distribution curves.


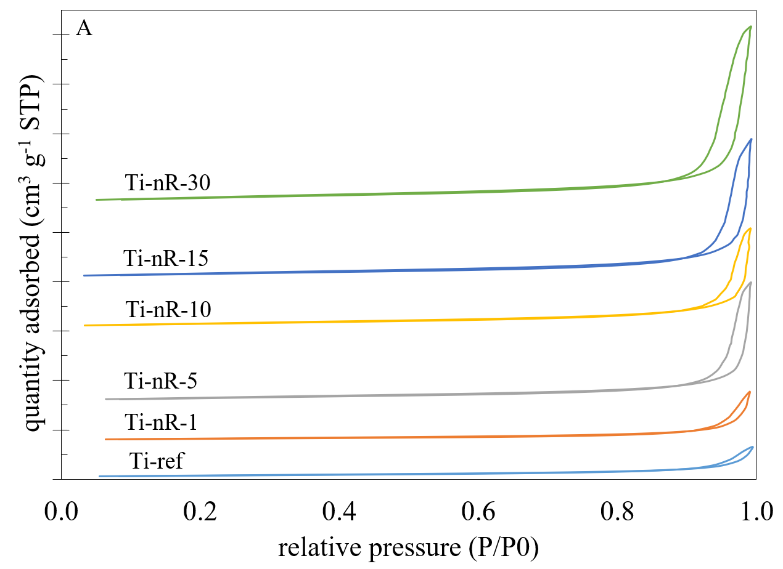

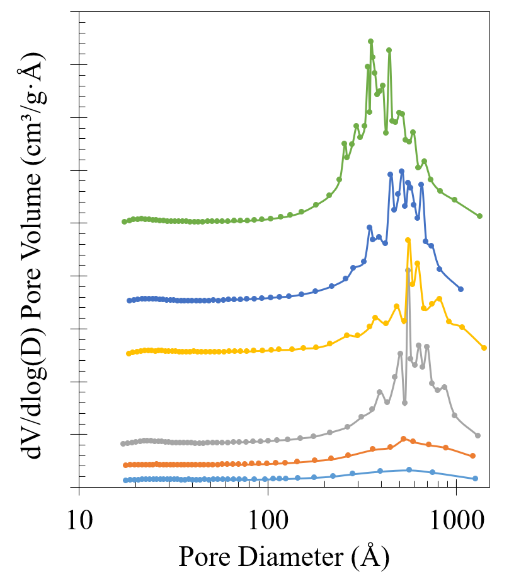


**Fig. S3** CO_2_ conversion and product selectivity over Ti-ref catalys


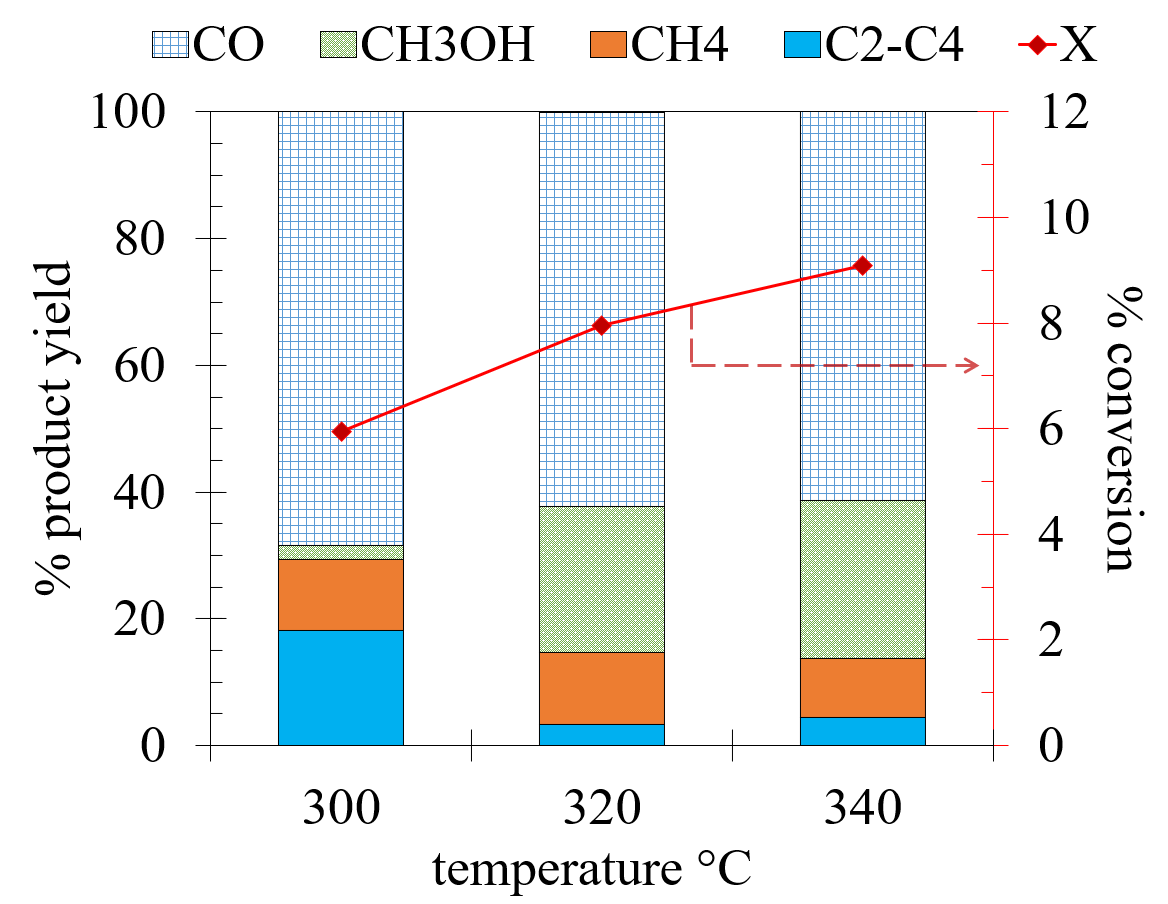


Table S1.- Raman vibrational modes assignment.

| **ref** | **Ti-nR-1** | **Ti-nR-2.5** | **Ti-nR-5** | **Ti-nR-10** | **Ti-nR-15** | **Ti-nR-30** | **Silva et al** | **Zhang et al.** | **Related to** |
| --- | --- | --- | --- | --- | --- | --- | --- | --- | --- |
| **-** | - | - | 127 | 130 | 130 | - | (Ag) - αNa_2_ | - | Na compounds |
| 144 | 145 | 145 | - | - | - | - | B1g | - | TiO_2_ anatase phase |
| 197 | 198 | 197 | - | - | - | - | Eg | - | TiO_2_ anatase pase |
| - | - | - | 203 | 205 | 205 | 202 | (Bg) - τO_1_-Ti_3_-O_2_ | - | Na_2_TiO_3_O_7_ |
| - | - | - | 278 | 277 | 279 | 279 | τO_5_-Ti_2_-O_6_ | Na-O-Ti | Na_2_TiO_3_O_7_ |
| 396 | 397 | 397 | - | - | - | - | B1g | - | TiO_2_ anatase phase |
| - | - | - | - | - | 418 | - | - | - | - |
| - | 444 | 444 | 445 | 431 | 462 | 454 | σO_5_-Ti_1_-O_4_ | Ti-O-Ti | TiO_2_ rutile phase |
| 517 | 517 | 517 | - | - | - | - | A1g | - | TiO_2_ anatase phase |
| 638 | 638 | 639 | 646 | 639 | 637 | 639 | Eg | Ti-O-Ti of TiO_6_ | TiO_2_ anatase phase |
| **-** | - | - | 701 | - | - | - | - | Ti-O-Ti of TiO_6_ | - |
| **-** | - | - | 902 | - | - | 910 | Ti-O bond | Ti-O associated  to Na ions | Na_2_TiO_3_O_7_ |

Table S2 Chemical composition results for Ti-nR-x samples from the ICP analysis.

| sample | Ti wt % | Na wt % | Ti/Na |
| --- | --- | --- | --- |
| Ti-ref | 60.9 | - | - |
| Ti-nR-1 | 55.5 | 8.4 | 6.6 |
| Ti-nR-5 | 44.1 | 10.7 | 4.1 |
| Ti-nR-10 | 43.3 | 11.3 | 3.8 |
| Ti-nR-15 | 42.6 | 11.8 | 3.6 |
| Ti-nR-30 | 42.1 | 12.0 | 3.5 |

Table S3 Steady state reaction rates for reactant and products as a function of reaction temperature.

|  |  | steady state reaction rates x10^-8^ mol*_i_* g_cat_^-1^ s^-1^ | | | |  |
| --- | --- | --- | --- | --- | --- | --- |
| Catalysts | T°C | CO | CH_4_ | C2-C4 | CH_3_OH | CO_2_ |
| Ti-nR-5 | 300 | 106.3 | 10.7 | 0.0 | 53.3 | 170.4 |
|  | 320 | 148.4 | 13.7 | 0.0 | 96.9 | 259.1 |
|  | 340 | 169.2 | 11.8 | 0.0 | 146.3 | 327.2 |
| Ti-nR-10 | 300 | 250.2 | 61.2 | 15.3 | 123.3 | 449.9 |
|  | 320 | 278.6 | 65.4 | 25.0 | 162.7 | 531.7 |
|  | 340 | 335.1 | 55.0 | 33.4 | 231.0 | 654.4 |
| Ti-nR-15 | 300 | 254.3 | 109.0 | 34.8 | 92.8 | 490.8 |
|  | 320 | 313.6 | 122.3 | 50.8 | 140.5 | 627.2 |
|  | 340 | 317.7 | 104.3 | 61.4 | 198.4 | 681.7 |
| Ti-nR-30 | 300 | 277.0 | 103.7 | 27.1 | 123.9 | 531.7 |
|  | 320 | 336.1 | 126.9 | 47.2 | 164.7 | 674.9 |
|  | 340 | 358.2 | 98.8 | 64.6 | 221.4 | 743.1 |
| Ti-Ref | 300 | 277.8 | 45.4 | 73.8 | 8.5 | 405.6 |
|  | 320 | 336.6 | 61.2 | 18.4 | 125.2 | 541.4 |
|  | 340 | 380.3 | 57.1 | 27.9 | 155.1 | 620.4 |
